# Contrasting effects of heat pulses on different trophic levels, an experiment with a herbivore-parasitoid model system

**DOI:** 10.1371/journal.pone.0176704

**Published:** 2017-04-28

**Authors:** Stijn J. J. Schreven, Enric Frago, Annemiek Stens, Peter W. de Jong, Joop J. A. van Loon

**Affiliations:** 1 Laboratory of Entomology, Plant Sciences Group, Wageningen University & Research, Wageningen, The Netherlands; 2 CIRAD Agricultural Research for Development, Saint-Pierre, La Réunion, France; Universidad de la Republica Uruguay, URUGUAY

## Abstract

Under predicted global climate change, species will be gradually exposed to warmer temperatures, and to a more variable climate including more intense and more frequent heatwaves. Increased climatic variability is expected to have different effects on species and ecosystems than gradual warming. A key challenge to predict the impact of climate change is to understand how temperature changes will affect species interactions. Herbivorous insects and their natural enemies belong to some of the largest groups of terrestrial animals, and thus they have a great impact on the functioning of ecosystems and on the services these ecosystems provide. Here we studied the life history traits of the plant-feeding insect *Plutella xylostella* and its specialist endoparasitoid *Diadegma semiclausum*, when exposed to a daily heat pulse of 5 or 10°C temperature increase during their entire immature phase. Growth and developmental responses differed with the amplitude of the heat pulse and they were different between host and parasitoid, indicating different thermal sensitivity of the two trophic levels. With a +5°C heat pulse, the adult parasitoids were larger which may result in a higher fitness, whereas a +10°C heat pulse retarded parasitoid development. These results show that the parasitoid is more sensitive than its host to brief intervals of temperature change, and this results in either positive or negative effects on life history traits, depending on the amplitude of the heat pulse. These findings suggest that more extreme fluctuations may disrupt host-parasitoid synchrony, whereas moderate fluctuations may improve parasitoid fitness.

## Introduction

Current climate change involves not only an increase in the average temperature, known as global warming, but also an increase in climatic variability [1], which will lead to more intense and more frequent heatwaves [2, 3]. These climatic changes have affected species distributions, they have disrupted species interactions, and they have ultimately triggered changes at the community and ecosystem level [4]. Relative to the effects of global warming, the effects of extreme events such as heatwaves are little understood. These extreme weather events (extreme temperature and precipitation events and storms), however, have been reported to underlie shifts in distribution and phenology of various invertebrate, vertebrate and plant species [5–7]. Studies that address the combined effects of increased mean temperature and extreme temperature events on species interactions are needed to reliably predict the consequences of climate change on species interactions, with potential effects on ecosystems and the services they provide to our society.

Studies on the responses of insects to climate change can significantly enhance our ability to predict the impact of climate change on ecosystems. Insects are the most diverse group of animals on land [8], and they fulfil many economically and ecologically important roles including nutrient cycling, pollination and pest control [9]. Insects are ectotherms, and hence their body temperature varies along with the ambient temperature. This means that temperature will affect their metabolism, physiology, and behaviour, and consequently their fitness [10]. As a response to global warming, the geographical distribution and life history of many insect species have changed. Changes in distribution mostly involve range shifts and expansions, whereas at the life history level, many species have advanced their activity in the season or increased the number of generations per year [11]. Additionally, as temperature increases, most insects attain a smaller adult body size (temperature-size rule) [12], probably because growth rate and overall development time have different thermal responses [13, 14]. In ectotherms, fitness generally increases with temperature to a maximum and then declines steeply [10, 13, 15, 16].

Insect responses can differ depending on whether the temperature regime changes in terms of variability or mean temperature [17, 18]. This has been observed when comparing the performance of insects developing under either constant or fluctuating thermal regimes [18]. With the same overall mean, insects often perform better under fluctuating temperatures, except when the range of fluctuating temperatures includes supra-optimal values [18]. Short exposure to temperatures above the optimum (i.e. a heat shock) can lead to slower development of immature stages [19] and reduced survival, longevity and fecundity in adults [20, 21]. The negative effects of thermal stress can carry over different life stages [19, 20] and they can even have a transgenerational effect (i.e. from the exposed adults to their offspring) [21].

How changes in life-history traits affect complex species interactions is still poorly understood [11, 22]. Comparing responses of different trophic levels may disentangle this complexity and may enable general predictions at the community level [23]. For example, Voigt and co-workers [24] showed that higher trophic levels are more sensitive to climatic variability and suggested this phenomenon could be widespread. Relative to plants, herbivorous insects show increased growth rate with increasing temperatures [23]. Natural enemies of these insects (in particular the more specialized endoparasitic wasps), are also affected by changes in temperature but their responses are constrained by the response of their herbivorous hosts [25]. For this reason, the negative impact of increased temperatures is often stronger in parasitoids than in their hosts. For example, increased temperatures can improve host resistance against parasitism, or they can eliminate important microbial symbionts that are used by the parasitoid for successful parasitism [26]. Such differential thermal sensitivity of hosts and parasitoids can potentially result in disruption of synchrony between these two trophic levels, and can ultimately lead to more frequent and intense pest outbreaks as climatic variability increases [27].

In this study, we compare the effects of different thermal regimes in a system composed of a plant, a herbivorous insect, the moth *Plutella xylostella* (L.) (Lepidoptera: Plutellidae), and one of its main natural enemies, the specialist parasitoid wasp, *Diadegma semiclausum* (Hellén) (Hymenoptera: Ichneumonidae). We exposed the immature stages of these insects to five different thermal regimes that varied in mean temperature, and in the amplitude of a heat pulse. In this study we define a heat pulse as a short-term (i.e. 4-hour) temperature increase. To assess the responses of the two insect species to our experimental treatments we measured a suite of life-history traits. *Plutella xylostella* is a worldwide pest of brassicaceous crops [28] and successfully develops over a wide range of constant temperatures (8 to 32°C) [29]. This insect can even survive when exposed to a wider range of temperatures if extreme temperatures are not constant (4:12 to 28:38°C during night:day) [29]. *Diadegma semiclausum* tolerates a narrower range of temperatures [30], with performance dropping at temperatures above 30°C [31]. Here we hypothesize that:

1. Increased mean temperatures will accelerate development and growth, and decrease adult body mass and survival of both the host and the parasitoid.
2. A heat pulse not exceeding the optimum temperature of the species will accelerate development and growth, whereas a heat pulse exceeding the optimum temperature will reduce survival and adult body mass and retard development and growth due to heat stress.
3. The temperature treatments imposed will have a differential effect on the two different trophic levels. The parasitoid will be more sensitive to temperature change than the host, and therefore will be more negatively affected by the heat pulses.

## Material and methods

### Insect rearing

*Plutella xylostella* and *Diadegma semiclausum* were obtained from our stock colonies, which originated from nearby field populations in Wageningen (The Netherlands) and which were established, respectively, in 2010 and 2011. *Plutella xylostella* and *D*. *semiclausum* were reared in separate climate rooms under constant conditions (22°C, 40–50% relative humidity, L16:D8) at the Laboratory of Entomology, Wageningen University, The Netherlands. Three-week-old Brussels sprouts plants (*Brassica oleracea* L. var. *gemmifera* cv. Cyrus) were used to feed the caterpillars in the experiments. In experiment 1, these were replaced by new ones when almost all leaf tissue was consumed or when plants started to show signs of drought stress.

### Experimental design

#### Experiment 1

We followed the development of *P*. *xylostella* and *D*. *semiclausum* from egg to adult under four thermal regimes in different climate cabinets (Elbanton B.V.). All thermal regimes had a day:night photoperiod of 16:8 h, corresponding to summer field conditions in The Netherlands. Night temperature was set at 5°C cooler than day temperature. Inside the climate cabinets, fluorescent tubes provided light during daytime. Additional LED lights (red 620–660 nm and blue 465–475 nm) were also placed at the top of the cabinet, to increase light intensity for photosynthesis and allow for normal plant growth. Two types of thermal regimes were applied: a regime with an alternating night and day temperature without heat pulse, and a regime aimed at emulating a heatwave. In this regime, temperature was increased +5°C for four hours five consecutive days every week. To emulate a more natural situation this increased temperature was applied when lights were on, approximately at midday (12:00 to 16:00). These two thermal regimes were applied to the insects at two different mean temperatures (20°C (night 15°C) and 25°C (night 20°C)) leading to a total of four different thermal regimes (Table 1). Two climate cabinets could be used at a time, with the different thermal regimes tested in two temporal blocks. The regimes with +5°C heat pulse were tested in one block, and the regimes without heat pulse in another. Relative humidity varied daily between 70–90% in the cabinet with the 20°C regimes and between 75–100% in the cabinet with the 25°C regimes.

**Table 1:** Temperature settings over a day for the different thermal regimes applied in experiments 1 and 2.

	Experiment 1				Experiment 2	
hour	20/15 + 0	20/15 + 5	25/20 + 0	25/20 + 5	25/20 + 0	25/20 + 10
0–7	15	15	20	20	20	20
7–12	20	20	25	25	25	25
12–16	20	(20) 25	25	(25) 30	25	(25) 35
16–23	20	20	25	25	25	25
23–0	15	15	20	20	20	20

Thermal regimes are expressed as “day temperature”/ “night temperature” + “heat pulse” (°C). In parentheses are the temperature settings on the two days per week when no heat pulse was applied.

To begin the experiment, *Plutella xylostella* eggs were incubated at each specific thermal regime within 24 hours after oviposition. During egg incubation, the heat pulse was not applied. Pairs of neonate larvae were placed on single plants in separate 0.72 L BugDorm containers (BugDorm, MegaView Science Co., Ltd. Taiwan; hereafter “container”). In total, 76–78 *P*. *xylostella* larvae paired in 38–39 containers were used per thermal regime. When larvae reached the second instar (L2), the pairs in 19–20 containers were parasitised by exposing them individually to an adult mated female of *D*. *semiclausum* in a small glass tube [32]. To assess parasitism we observed whether the wasp inserted her ovipositor into the larva. The larva was then placed back on the plant. Containers thus included a plant with either two parasitised or two unparasitised larvae. Performance and fate of the non-parasitised and parasitised larvae was subsequently monitored (described below under “Variables measured”). Parasitism failed in 12 individuals (i.e. a host pupa developed) out of a total of 156 within the four regimes. Three *D*. *semiclausum* adults were excluded from analyses because they were found dead and thus we were unable to measure their fresh adult weight and record their date of adult eclosion.

#### Experiment 2

As in experiment 1, we followed egg-to-adult development of hosts and parasitoids, but this time testing the effect of exposure to a more extreme heat pulse. One combination of day and night temperature was tested (20°C night and 25°C day temperature) with or without an additional +10°C increase on top of day temperature from 12:00 to 16:00 for five consecutive days a week (Table 1). Photoperiod (and timing of day and night temperature) was the same as in Experiment 1, but compared to the first experiment different climate cabinets (Snijders Scientific, type ECD01E) without LED supplementation were used. The experiment was conducted over six serially replicated temporal blocks. In all blocks the regimes with and without heat pulse were placed in independent cabinets.

Eggs of *P*. *xylostella* were incubated at a thermal regime of 20°C night and 25°C day temperature within 24 h after oviposition. Two neonate larvae were placed on each of the plants in the climate cabinet. Larvae were parasitised as in Experiment 1. One day after parasitism, one larva was removed, leaving one larva per plant. When larvae reached the pupal stage, they were maintained in the climate cabinet, but each one was individually placed in a tube, which was closed up with a cotton plug. In total, 55–62 individuals were tested per species and thermal regime.

### Variables measured

For each individual insect, survival was checked on a daily basis. The date of adult eclosion was recorded and freshly eclosed adults were killed (experiment 1, using ethyl acetate; experiment 2, using freezing at -18°C for at least 2 hours) in order to determine their sex, and to measure their fresh adult body mass using a micro-electrobalance (Experiment 1: Mettler-Toledo NewClassic MF ML54, Mettler-Toledo B.V. Netherlands; Experiment 2: Sartorius CP2P, Sartorius AG Germany). For *D*. *semiclausum*, development time was measured counting from the date of parasitisation. Growth rate was calculated as the logarithm of fresh adult body mass, divided by the time from egg to adult eclosion [33].

### Statistical analyses

All analyses were performed in R (R 3.0.2 http://www.r-project.org/). Adult body mass, development time and growth rate were analysed by fitting a linear model (i.e. a Gaussian error distribution) with the glm function. Survival was tested with a generalized linear model with the glm function but assuming a binomial error distribution (each individual was considered as reaching adulthood or not). The quasibinomial error distribution was used to prevent overdispersion. In these models the following categorical fixed factors were included: species (either the parasitoid or the host lepidopteran), the mean temperature treatment and the heat pulse treatment. In these models, sex was also included as fixed predictor, except in the models for survival where sex could not be determined in those individuals that died before adulthood. We also included as predictors interaction terms between species and the two temperature treatments. Significant interactions would represent varying effects of the treatments on the two species studied, our predicted *a priori* hypothesis. In these models model reduction was applied by removing non-significant interactions (P-value < 0.05). Tukey *post-hoc* comparisons (Bonferroni adjusted) were done on simpler models within species and sex, using the glht function.

## Results

### Experiment 1: Increased mean temperature and a +5°C heat pulse

Under the different thermal regimes tested, host and parasitoid survival rate ranged from 18–55% and 34–44%, respectively (Table 2). Survival of the parasitoid and its host was not affected by the mean temperature to which they were exposed (GLM, t = -1.195; df = 1, 290; P = 0.2329; Table 3). The heat pulse, however, affected the survival of both species in a different manner as revealed by the significant interaction between this treatment and species identity (species: t = 1.964; df = 1, 290; P = 0.0505; heat pulse: t = 0.010; df = 1, 290; P = 0.9918; interaction species x heat pulse: t = -2.249; df = 1, 290; P = 0.0252). Relative to the parasitoid, under the heat pulse treatment the host had a higher survival (Table 2).

**Table 2:** Survival rate (%) of *P*. *xylostella* and *D*. *semiclausum* under the different temperature treatments in experiments 1 and 2.

	Experiment 1				Experiment 2	
	20/15 + 0	20/15 + 5	25/20 + 0	25/20 + 5	25/20 + 0	25/20 + 10
*Plutella xylostella*	40.0	55.3	18.4	55.3	47.3	88.7
*Diadegma semiclausum*	44.1	36.8	34.3	41.7	50.9	88.5

Thermal regimes as defined in Table 1.

**Table 3:** Generalized linear model coefficients for life history traits of *P*. *xylostella* and *D*. *semiclausum* in experiment 1.

	Survival				Development time				Adult body mass				Growth rate			
	estimate	SE	t	P	estimate	SE	t	P	estimate	SE	t	P	estimate	SE	t	P
Intercept	-0.300	0.266	-1.127	0.2608	20.373	0.235	86.721	**< 0.0001**	0.933	0.057	16.330	**< 0.0001**	0.0465	0.0035	13.258	**< 0.0001**
sex					0.093	0.168	0.551	0.5830	-0.377	0.044	-8.593	**< 0.0001**	-0.0191	0.0025	-7.594	**< 0.0001**
species	0.658	0.335	1.964	0.0505	2.217	0.210	10.549	**< 0.0001**	0.558	0.050	11.234	**< 0.0001**	0.0216	0.0036	5.972	**< 0.0001**
temp	-0.291	0.243	-1.195	0.2329	-6.288	0.212	-29.672	**< 0.0001**	-0.013	0.038	-0.347	0.7295	0.0117	0.0031	3.768	**0.0003**
pulse	0.004	0.346	0.010	0.9918	0.645	0.151	4.284	**< 0.0001**	-0.258	0.054	-4.794	**< 0.0001**	-0.0149	0.0031	-4.831	**< 0.0001**
species:temp					-0.725	0.290	-2.495	**0.0140**					0.0109	0.0043	2.544	**0.0123**
species:pulse	-1.096	0.487	-2.249	**0.0252**					0.208	0.077	2.691	**0.0082**	0.0105	0.0045	2.348	**0.0207**

Reference categories for the factors are: sex = female; species = *Diadegma semiclausum*; temp = 20°C day temperature; pulse = with +5°C heat pulse. Coefficients for adult body mass are based on the log-transformed data. Statistics are only given for factors and interaction terms included in the model for that dependent variable. Significant P-values (P < 0.05) are in bold.

**Fig 1:**
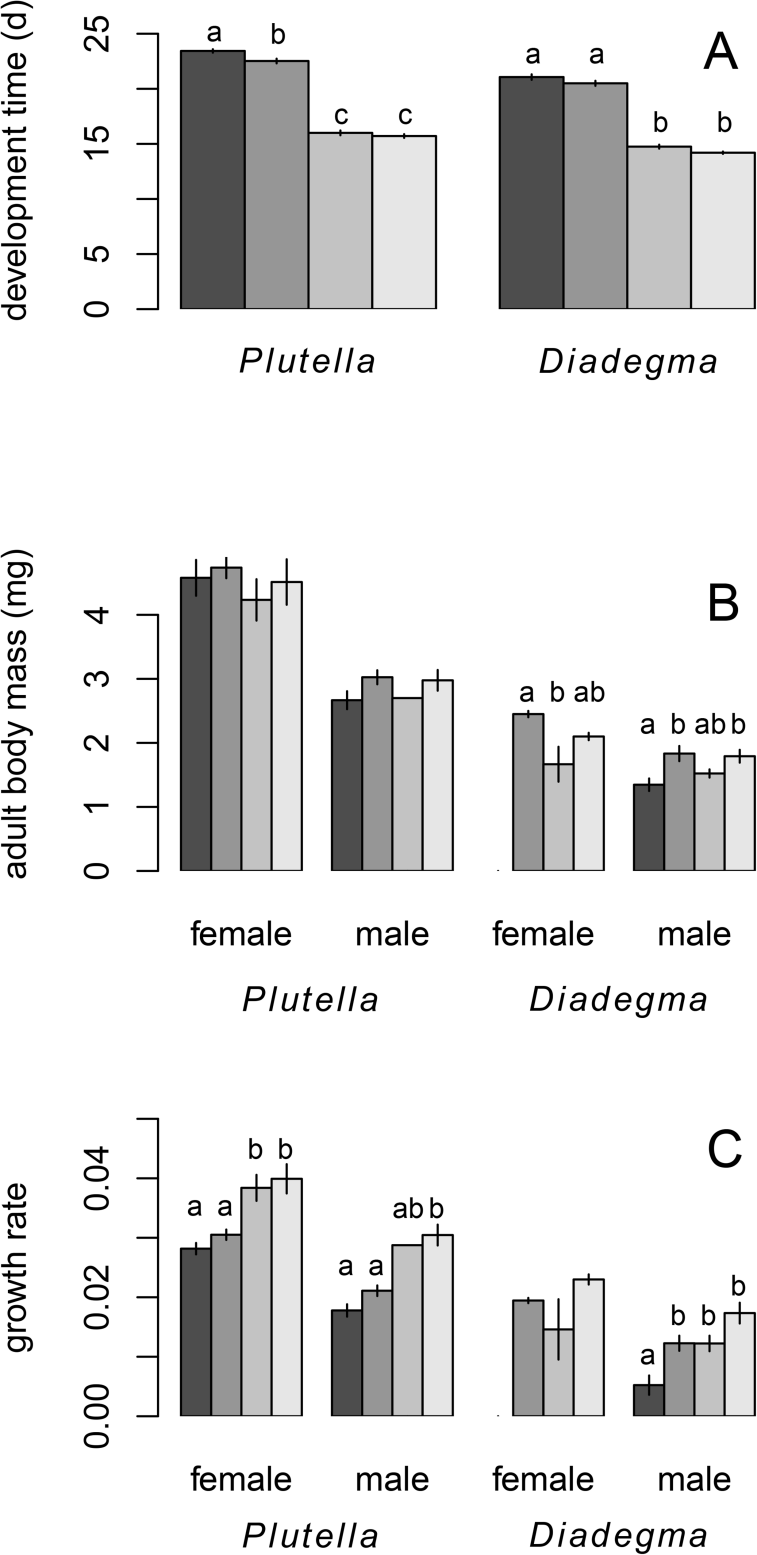
Life history traits (mean ± SE) of *Plutella xylostella* and *Diadegma semiclausum* exposed to different thermal regimes in experiment 1: A) development time, B) adult body mass, and C) growth rate. In B) and C) bars for females (f) and males (m) are displayed separately. Legend: the grey shades from dark to light indicate the thermal regimes 20/15+0, 20/15+5, 25/20+0 and 25/20+5 (as defined in Table 1), respectively. In A) males and females have been pooled since sex had no significant effect on development time. Different letters within the same insect species or within a combination of species and sex, indicate means that differ significantly, after Tukey *post-hoc* test.

The parasitoid and its host had a significantly different development time (t = 10.549; df = 1, 111; P < 0.0001), and this trait was not affected by the sex of the individuals (t = 0.551; df = 1, 111; P = 0.5830). Mean temperature affected both species differently as revealed by the significant interaction between species identity and this treatment (temperature: t = -29.672; df = 1, 111; P < 0.0001; interaction species x temperature: t = -2.495; df = 1, 111; P = 0.0140). Both the host and the parasitoid developed faster at the higher mean temperature regime, but for *P*. *xylostella* the decrease in development time with increasing mean temperature was larger than for *D*. *semiclausum* (Fig 1A). Heat pulse, on the other hand, had a similar effect on both species as the interaction with species identity was not significant (heat pulse: t = 4.284; df = 1, 111; P < 0.0001; the interaction term species x heat pulse was excluded from this model based on our model reduction approach that removed non-significant interaction terms). For both species, the heat pulse decreased mean development time.

Adult fresh body mass was significantly influenced by sex (t = -8.593; df = 1, 111; P < 0.0001), with females developing into larger individuals. Mean temperature, however, did not affect it (t = -0.347; df = 1, 111; P = 0.7295). The heat pulse significantly affected adult weight in a species-specific manner (species: t = 11.234; df = 1, 111; P < 0.0001; heat pulse: t = -4.794; df = 1, 111; P < 0.0001; interaction species x heat pulse: t = 2.691; df = 1, 111; P = 0.0082). Relative to their host, parasitoids attained a higher body mass when developing under a thermal regime with +5°C heat pulse (Fig 1B).

Growth rate was significantly influenced by sex (t = -7.594; df = 1, 111; P < 0.0001) with females showing an increased rate (Fig 1C). Both mean temperature and the heat pulse affected growth rate (temperature: t = 3.768; df = 1, 111; P = 0.0003; heat pulse: t = -4.831; df = 1, 111; P < 0.0001), but this effect depended on the species as revealed by the significant interaction between the two thermal treatments imposed and species identity (species: t = 5.972; df = 1, 111; P < 0.0001; interaction species x temperature: t = 2.544; df = 1, 111; P = 0.0123; interaction species x heat pulse: t = 2.348; df = 1, 111; P = 0.0207). The host had a higher growth rate in response to increased mean temperature, compared to the parasitoid. Relative to their host, growth rate was higher for parasitoids when they developed at a thermal regime with +5°C heat pulse (Fig 1C).

### Experiment 2: Heat pulse of +10°C

In the second experiment, survival rates varied between 47–89% and 51–89%, respectively, for the host and the parasitoid (Table 2). Both species had similar survival (t = -0.297; df = 1, 229; P = 0.7670; Table 4), but under the heat-pulse regime they had increased survival (t = -6.066; df = 1, 229; P < 0.0001).

**Table 4:** Generalized linear model coefficients for life history traits of *P*. *xylostella* and *D*. *semiclausum* in experiment 2.

	Survival				Development time				Adult body mass				Growth rate			
	estimate	SE	t	P	estimate	SE	t	P	estimate	SE	t	P	estimate	SE	t	P
Intercept	2.101	0.331	6.349	**< 0.0001**	15.477	0.120	129.368	**< 0.0001**	0.68333	0.03360	20.337	**< 0.0001**	0.0447	0.0022	20.11	**< 0.0001**
sex					0.007	0.118	0.056	0.9551	-0.38530	0.03503	-10.998	**< 0.0001**	-0.0247	0.0023	-10.67	**< 0.0001**
species	-0.095	0.319	-0.297	0.7670	-0.081	0.144	-0.565	0.5727	0.74326	0.03485	21.324	**< 0.0001**	0.0473	0.0023	20.50	**< 0.0001**
pulse	-2.090	0.345	-6.066	**< 0.0001**	-0.445	0.174	-2.561	**0.0114**	0.07104	0.03689	1.926	0.0560	0.0041	0.0024	1.67	0.0970
species:pulse					0.930	0.248	3.751	**0.0002**								

Reference categories for the factors are: sex = female; species = *Diadegma semiclausum*; pulse = with +10°C heat pulse. Coefficients for adult body mass are based on the log-transformed data. Statistics are only given for factors and interaction terms included in the model for that dependent variable. Significant P-values (P < 0.05) are in bold.

**Fig 2:**
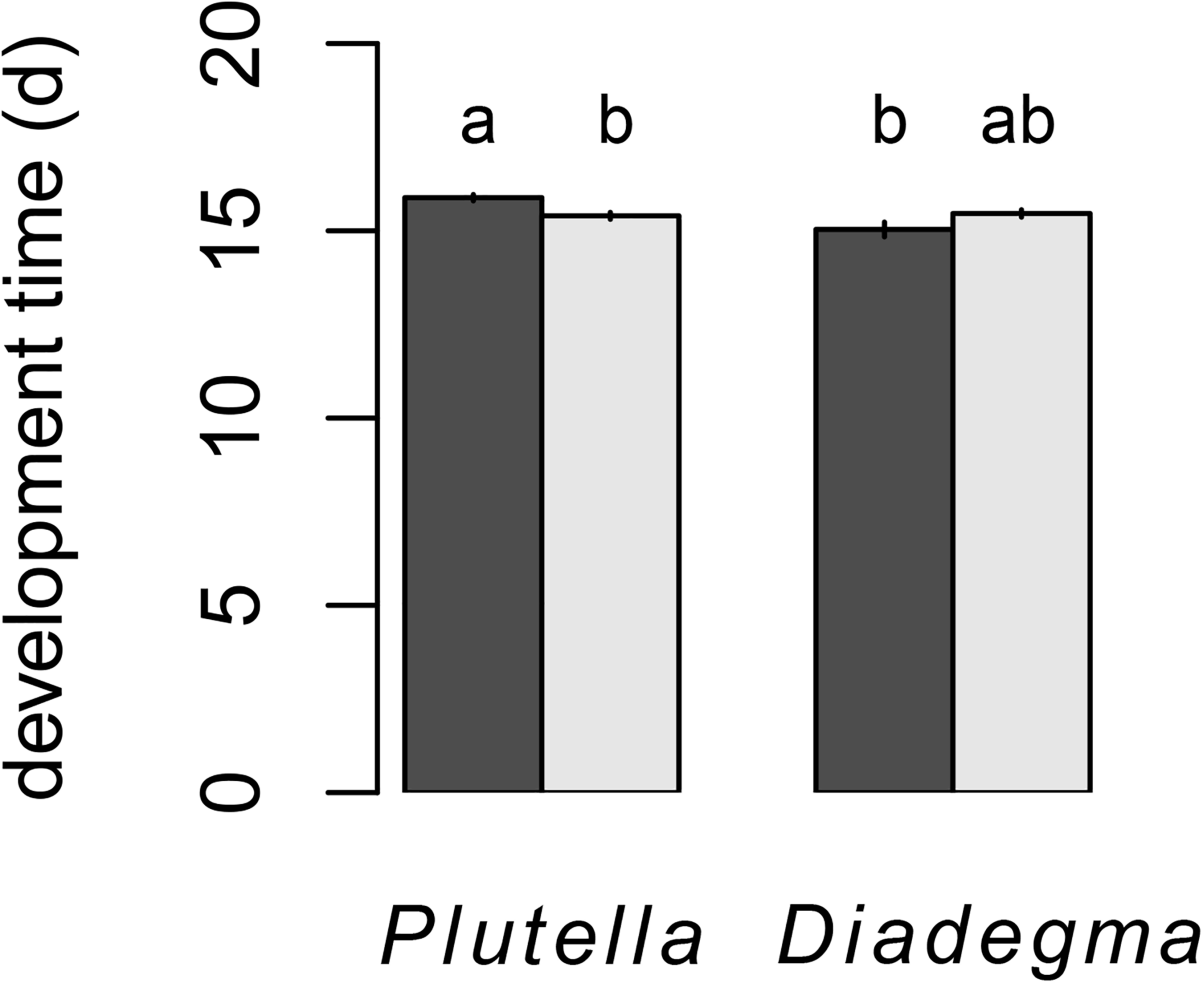
Development time (mean ± SE) of *Plutella xylostella* and *Diadegma semiclausum* under thermal regimes without (dark grey) and with + 10°C heat pulse (light grey). Data on males and females have been pooled since sex had no significant effect on development time. Different letters indicate means that differ significantly (Tukey *post-hoc* test).

Development time was not significantly affected by the sex of the individual (t = 0.056; df = 1, 156; P = 0.9551). Both species were differentially affected by the heat pulse treatment as revealed by the significant interaction term (species: t = -0.565; df = 1, 156; P = 0.5727; heat pulse: t = -2.561; df = 1, 156; P = 0.0114; interaction species x heat pulse: t = 3.751; df = 1, 156; P = 0.0002). When exposed to a heat pulse, the host developed faster than the parasitoid (Fig 2).

Adult fresh body mass was significantly influenced by the sex of the individual (t = -10.998; df = 1, 156; P < 0.0001) and both species had different adult body weights (t = 21.324; df = 1, 156; P < 0.0001). Females and the host developed into larger individuals, and these variables were not altered by the heat pulse treatment (t = 1.926; df = 1, 156; P = 0.0560).

As with adult body mass, growth rate was significantly influenced by sex (t = -10.670; df = 1, 156; P < 0.0001) and species (t = 20.500; df = 1, 156; P < 0.0001), but not by the imposed treatment (t = 1.670; df = 1, 111; P = 0.0970). Males and the parasitoid had smaller growth rates.

## Discussion

This study shows that different life history traits respond in a different way to changes in temperatures, and that these traits are differentially affected by either a heat pulse or a mean increase in temperature. The response also depends on the amplitude of the heat pulse, and most importantly on the species. As predicted by our *a priori* hypothesis the host and the parasitoid differed in their sensitivity to temperature variability. These differences can be caused by a direct effect of the temperature on the insects, an indirect effect via the quality of the plant, or a cascading effect on the parasitoid via changes in the quality of their host [32, 34]. Direct effects of temperature on the herbivore probably overrule plant-mediated effects [34] whereas the relative importance of the direct and indirect effects of temperature on the parasitoid remains to be elucidated [25, 32, 35].

Relative to the constant day temperature regimes, development time of *P*. *xylostella* was shorter when the insects were experimentally exposed to heat pulses of +5 and +10°C. Growth rate of *P*. *xylostella*, was higher at the 25°C regime compared to the 20°C regime, but did not differ between treatments with or without the heat pulse. This suggests that development time is more sensitive to temperature fluctuations than growth rate. This is in line with previous findings that suggest that development time has a stronger dependence on temperature than growth rate [13, 14].

For *D*. *semiclausum*, the +5°C heat pulse led to shorter development time, whereas the +10°C heat pulse had the opposite effect. When exposed to a +5°C heat pulse, growth rate increased and adults reached a larger body mass. This could be caused by altered host regulation by the parasitoid [36], or through an effect of temperature on host physiology. The heat pulse can therefore lead to a host of higher quality for the parasitoid, or it can allow the parasitoid larva to have an increased efficiency in assimilating the biomass of the host [32]. The fact that *D*. *semiclausum* took longer to reach adulthood at the +10°C heat pulse, indicates that the optimal temperature for development time of *D*. *semiclausum* is below the highest temperature reached in this regime (i.e. 35°C) (as in Fig 1 of Colinet and co-workers [18]). This is in agreement with previous findings on the temperature tolerance range of *D*. *semiclausum* [30, 31, 37].

Our results also show that the amplitude of the heat pulse influenced the measured traits. *Diadegma semiclausum* had a longer development time when exposed to the +10°C heat pulse, which is often considered a negative effect because insects are exposed to natural enemies for a longer period of time [38, 39]. The +5°C heat pulse, however, had a positive effect on parasitoids as they developed into larger adults that reached adulthood faster. This supports previous work that suggests that extremely high temperatures usually have a negative effect on insects [21, 40, 41], whereas moderate fluctuations may have a neutral or a beneficial effect on them [40, 42].

The two species differed in their thermal sensitivity as revealed by life history trait responses when both the parasitoid and its host were exposed to heat pulses. *Diadegma semiclausum* was more sensitive to temperature variability than *P*. *xylostella*, with negative consequences when the heat pulse was +10°C, but positive effects when the pulse was +5°C. This supports the general hypothesis that higher trophic levels may be more sensitive to variability in climate factors [24]. However, results of contrasting directions underlie this hypothesis: effects can be positive or negative for the parasitoid, as found in our experiments. It has been previously suggested that parasitoids are more vulnerable to extreme temperatures for several reasons. The response of the parasitoid may be constrained by that of the host, or increased temperatures can improve host resistance. Microbial players can also be relevant, for example if extreme heat kills symbiotic microorganisms that the parasitoid uses for successful parasitism [25]. Similarly, the more extreme heat pulse used in our study negatively affected the parasitoid, i.e. slowing down development, relative to the host. Under a global change scenario, such differences between parasitoids and their hosts may disrupt the synchronisation of their phenology and can lead to increased frequency of pest outbreaks due to a failure of pest suppression by natural enemies [25]. However, the consequences of increased thermal variability can be system- and species-specific [43]. In some systems, the third trophic level benefits from increased temperatures, whereas the herbivore suffers from them [43–45]. In others this response is reversed [46] or there is little or no change [47, 48].

The relationship between climate change and host-parasitoid communities is therefore of great interest, not only to assess the impact of climate change on natural ecosystems, but also to make predictions on pest outbreaks in agricultural systems and managed forests [49]. In our study system, it is likely that with more extreme fluctuations (peaks of 35°C or more) *D*. *semiclausum* development may be delayed to such a degree that it misses the window of opportunity to parasitise its host. *Plutella xylostella* is an important agricultural pest [28]. If *D*. *semiclausum* does not adapt to the new conditions, or the pest is not controlled by other natural enemies, biological control of *P*. *xylostella* may be jeopardised under a global change scenario. With moderate fluctuations (+5°C) above daily temperature, *D*. *semiclausum* may benefit by increased body mass and consequently increased fitness [50] and *P*. *xylostella* control may thus be more effective. Our study did not measure adult longevity and fecundity [51–53], and future studies need to measure these traits in order to accurately estimate the fitness consequences of the heat pulses. Additionally, since different life stages can be differentially sensitive [54, 55], the timing of such fluctuations in the lifecycle of insects will influence the effects on fitness. The complex interactions between these factors need to be investigated in the future for a more comprehensive view on climate variability and its effects at the species and ecosystem level.
